# Atrophy of primary lymphoid organs induced by Marek’s disease virus during early infection is associated with increased apoptosis, inhibition of cell proliferation and a severe B-lymphopenia

**DOI:** 10.1186/s13567-018-0526-x

**Published:** 2018-03-27

**Authors:** Camille Berthault, Thibaut Larcher, Sonja Härtle, Jean-François Vautherot, Laetitia Trapp-Fragnet, Caroline Denesvre

**Affiliations:** 1 ISP, INRA, Université Tours, 37380 Nouzilly, France; 2 APEX, INRA, ONIRIS, ENVN, 44307 Nantes, France; 3 Department of Veterinary Science, Ludwig-Maximilians University of Munich, 80539 Muenchen, Germany

## Abstract

Marek’s disease is a multi-faceted highly contagious disease affecting chickens caused by the Marek’s disease alphaherpesvirus (MDV). MDV early infection induces a transient immunosuppression, which is associated with thymus and bursa of Fabricius atrophy. Little is known about the cellular processes involved in primary lymphoid organ atrophy. Here, by in situ TUNEL assay, we demonstrate that MDV infection results in a high level of apoptosis in the thymus and bursa of Fabricius, which is concomitant to the MDV lytic cycle. Interestingly, we observed that in the thymus most of the MDV infected cells at 6 days post-infection (dpi) were apoptotic, whereas in the bursa of Fabricius most of the apoptotic cells were uninfected suggesting that MDV triggers apoptosis by two different modes in these two primary lymphoid organs. In addition, a high decrease of cell proliferation was observed from 6 to 14 dpi in the bursa of Fabricius follicles, and not in the thymus. Finally, with an adapted absolute blood lymphocyte count, we demonstrate a major B-lymphopenia during the two 1st weeks of infection, and propose this method as a potent non-invasive tool to diagnose MDV bursa of Fabricius infection and atrophy. Our results demonstrate that the thymus and bursa of Fabricius atrophies are related to different cell mechanisms, with different temporalities, that affect infected and uninfected cells.

**Electronic supplementary material:**

The online version of this article (10.1186/s13567-018-0526-x) contains supplementary material, which is available to authorized users.

## Introduction

Marek’s disease (MD) is a major disease of poultry, with an estimated annual cost of 1–2 billions of dollars [1]. Marek’s disease Virus (MDV) (or *Gallid herpesvirus* type 2), caused by the highly contagious alphaherpesvirus, is mostly recognized for lethal T cell lymphoma and immunosuppression [2–4]. MDV has a tropism for immune cells in vivo, in particular B and T lymphocytes as well as macrophages [2, 4, 5]. Upon entry via the respiratory tract, MDV is transported to the major lymphoid organs of birds (bursa of Fabricius which will later be called bursa for the sake of clarity, thymus and spleen) in which it replicates, causing an early cytolytic infection in B and T-cells, between 3 and 7 days post-infection (dpi) [2, 5, 6]. Subsequently, between 7 and 10 dpi, MDV establishes latency in CD4^+^ T-cells. A small number of these cells will be transformed leading to a mono- or oligoclonal T-cell lymphoma [7]. During the 1st week of infection, MDV reaches the feather follicle epithelium of the skin, from which MDV is persistently excreted into the environment where it remains infectious for weeks [8–11].

The early cytolytic infection is associated with an immunosuppression which increases the susceptibility of infected birds to other infectious agents, like *E. coli* or coccidiosis [12, 13]. It also reduces the response to vaccines, e.g. against infectious bronchitis virus and *Mycoplasma synoviae* or model antigens, such as tetanus toxoid [12, 14, 15]. MDV early infection is also associated with an early and transient atrophy of the bursa and thymus, which is more or less severe depending on the virulence of the strain [16]. For each organ, the atrophy is accompanied by major histological lesions, a loss of thymic cortical cells and a degeneration of bursa follicles [17, 18]. The thymus and bursa are the primary lymphoid organs of chickens, where T- and B-cells undergo development, respectively. After maturation, naive lymphoid cells exit into the blood and reach the secondary lymphoid organs (e.g. the spleen) and peripheral tissues. Differentiated T-cells are detectable out of the thymus in the embryo from stages E15–E16 [19] and B-cells expressing IgM start to emigrate out of the bursa around hatching [20].

Because of its capacity to directly invade and replicate in the thymus and the bursa of newly-hatched chicks for a short period of time, MDV is particularly interesting for the study of the mechanisms leading to primary lymphoid organ atrophy and the consequences on the immune response. The peak of atrophy in the thymus and bursa was shown to occur around 10–14 dpi, when MDV lytic antigens are usually no longer detectable in these tissues. Baigent et al. detected the MDV lytic protein pp38 by immunochemistry and cytometry in cells of the medulla of the thymus and of bursa follicles in susceptible newly-hatched birds infected with HPRS-16 from 4 to 6 dpi [21]. Surprisingly, they estimated that less than 0.1% of the cells were pp38 positive, indicating that death of infected cells alone cannot explain the extent of the subsequent atrophy. Moreover, the presence of virus particles was visualized by transmission electron microscopy at 7 dpi in the thymus of chicks infected with HPRS-16, usually in immature lymphocytes and exclusively in the medulla [22]. Several years ago, Morimura et al. suggested that thymic atrophy was due to a transient apoptosis of thymocytes induced by MDV. Indeed, they detected a high level of apoptosis by DNA laddering in explanted thymocytes of vvMd5-infected birds at 1 week post-infection (pi) and an increase of the number of macrophages engulfing apoptotic cells in the thymic cortex by histology, a feature which was not found at 2 weeks pi [18].

Various pathogens of mammals and birds invading the thymus were shown to induce thymus atrophy, including viruses, for e.g. human immunodeficiency virus (HIV), human cytomegalovirus, measles virus, mouse hepatitis virus, murine leukemia virus, mouse lymphocytic choriomeningitis virus, a new mouse ß-herpesvirus and chicken anemia virus (CAV) [23–25]. Different molecular mechanisms have been reported leading to this thymic atrophy: a direct lytic infection of thymocytes leading to their death, apoptosis induction of uninfected cells by viral products, alteration of thymic microenvironment [notably by infection of thymus epithelial cells (TEC) or dendritic cells (DC)] resulting in their death or modulation of cell functions, as well as an increase in systemic or local factors such as glucocorticoids and pro-inflammatory mediators (reviewed in [24]). Also, thymic atrophy was occasionally associated with a severe lymphopenia, as reported for highly pathogenic avian influenza virus in humans [26]. Several bird-specific viruses infect the bursa, among which infectious bursal disease virus (IBDV) and velogenic Newcastle disease virus (NDV). Both induce a marked degeneration of lymphocytes in the medullary region of the bursa [27, 28] and an increased apoptosis in the bursa [29–31].

In this study, our aim was to explore more deeply the cellular mechanisms leading to primary lymphoid organ atrophy in specific pathogen-free (SPF) susceptible birds. We examined apoptosis induction in the thymus and bursa and we also investigated whether other cellular processes could be involved in organ atrophy. Moreover, we aimed at studying if infected cells were the only targets of cell modifications or if uninfected cells were also affected. We show that cell death by apoptosis is increased in the thymus, but also in the bursa. In addition, we demonstrate that, in contrast to the thymus, apoptosis essentially affects uninfected cells in the bursa. We also observed an inhibition of cell proliferation in the bursa of infected birds that lasted longer than the cytolytic infection. Lastly, we show by blood count a strong B-lymphopenia during MDV early infection, which was associated with the primary lymphoid organ atrophy. Therefore, blood count might become a promising method to monitor and predict MDV-induced bursa atrophy.

## Materials and methods

### Viruses

The very virulent strain (vv) RB-1B [16, 32–34], which is known to induce lymphoid organ atrophy in chickens lacking maternal antibody [16] was amplified in culture on chicken embryonic skin cells (CESC) as previously described [35]. Briefly, CESC were obtained from 12-day-old embryos digested by collagenase (0.2 mg/mL) for 20 min. Cells in suspension were then rinsed and cultivated on plastic dishes pre-coated with gelatin. The non-adherent cells, mostly red-blood cells were washed before use. Herein CESC were derived from the SFP Brown Leghorn (LD1) INRA chicken line. The virus stock used for the in vivo experiment did not exceed one passage in culture after bird explantation. The virus titer was of 2.8 × 10^4^ pfu/mL on CESC.

### Antibodies

Anti-CD4 (clone CT4), anti-CD8-FITC (clone CT8), anti-Bu1 (clone AV20) were obtained from Clinisciences (Nanterre, France). The thrombocyte/monocyte marker mAb K1 [36] and anti-CD45 mAb (clone 16-6) were kindly provided by Prof. B. Kaspers (Institute for Animal Physiology, University of Munich). Conjugations of mAb K1 and mAb CT4 to RPE, mAb 16-6 to APC and mAb AV20 to PerCP-Cy5.5 were performed using the respective Lynx Rapid Conjugation Kits^®^ according to the manufacturer’s instructions (AbD Serotec, Kidlington, UK). Prior to use, each coupled mAb was titrated individually on blood cells to determine the optimal staining dilution.

Polyclonal rabbit anti-PCNA antibody (#AHP1419, AbD Serotec) recognizing the proliferating cell nuclear antigen, was used to determine whether the cell proliferation in the thymus and the bursa is impacted by MDV infection. Monoclonal antibodies directed against MDV lytic antigens VP22, ICP4 and gB were previously described [35, 37] and the monoclonal antibody directed against the oncogenic Meq viral protein is described below. Two goat anti-mouse (GAM) secondary antibodies were used: GAM-Alexa 488 (IgG) (#A-11001, Thermo Fisher Scientific) and GAM-Alexa 488 F(ab’)^2^ (#A-11017, Thermo Fisher Scientific).

### Generation of an anti-Meq mouse monoclonal antibody and its characterization

The sequence encompassing nucleotides 307–1020 of the meq (MDV EcoRI Q) gene was amplified by PCR from pBK-MEQ-CMV plasmid generously donated by Prof. B. B. Kaufer (Institut für Virologie, Freie Universitaet Berlin, Germany) and sub-cloned with cMyc tag sequence in 5′ in the pFastBac™Dual (Thermo Fisher Scientific). The cMycMeq sequence was next transposed in the baculovirus bacterial artificial chromosome (BAC) in DH10 BAC according to the manufacturer’s instruction (Invitrogen). One positive BAC mini-preparation was transfected into Sf9 insect cells using lipofectamine 2000 (Thermo Fisher Scientific). Cell-free supernatant containing the recombinant baculovirus Bac-cMycMeq was harvested 6 days post-transfection before the transfected/infected cells were pelleted and spotted on poly-lysine (Sigma) coated coverslips to verify baculovirus replication with an anti-cMyc antibody or an anti-baculovirus antibody (monoclonal antibody G15—J. F Vautherot, data not shown). Baculovirus Bac-cMycMeq resulting from the transfection was passaged on Sf9 for inoculum production and cloned. A 1% NP40 insoluble extract from Bac-cMycMeq (clone 7) infected Sf9 cells was used to immunize C57Bl6 mice with QuilA (15 µg/injection) as an adjuvant (Protocol Number APAFIS#2718-201511061612253 v2). Immunization and fusion procedures were essentially as described previously [35]. The screening for Meq specific monoclonal antibodies secreted by hybridomas was performed by indirect immunofluorescence microscopy on fixed Sf9 cells infected with Bac-cMycMeq or with a baculovirus expressing gC used as a negative control. Subsequently, the selected monoclonal antibody, named Lamba7 (IgG2b isotype), was characterized on various cellular substrates (see Additional file 1): ESCDL-1 avian cells [38] transfected with the pBK-MEQ-CMV plasmid or infected with rRB-1B pp38mcherry (a kind gift of Prof. V. Nair, Avian Oncogenic Virus Group, The Pirbright Institute, UK), MSB-1 T-cell line, DT40 B-cell line (as a negative control) and MDV-induced tumors (of kidney or ovarian origin).

### In vivo experiments

Specific pathogen-free (SPF) and HVT/MDV maternal antibody-free White Leghorn chicks (B19/B19 haplotype) were used and obtained from INRA animal facilities. Note that these birds were specified Chicken Anemia Virus-free. Two groups of 20 2-day-old chicks (MDV-infected and mock-infected, named control, CTL) were constituted and housed in two different isolation units. The infected group (MDV) was inoculated intramuscularly with 2000 pfu (0.2 mL) of the RB-1B strain whereas the control group was inoculated with 2 × 10^5^ non-infected CESC. This injection route was chosen in agreement with the previous studies of Morimura et al. [18, 39]. Five or six, six and eight chicks were sampled for each group at 6, 10 and 14 dpi, respectively. At each time point, animals were blood sampled, euthanized, weighted and necropsied to collect lymphoid organs (bursa, thymus, and spleen). The relative lymphoid organ weight was determined by calculating the ratio of the weight of the organ (mg) divided by the body weight of the bird (g) multiplied by 100. Subsequently, samplings of each lymphoid organ were prepared and preserved before use to perform histology, cryosections and real-time PCR analysis as follows: (i) for histology, tissues were fixed in 4% formalin and included in paraffin in the next month, (ii) for cryosections, tissues were frozen directly in cold isopentane vapors and next conserved at −80 °C, (iii) for real-time PCR, tissues were directly frozen at −80 °C until DNA extraction and extracted DNA were subsequently stored at 4 °C until qPCR. Blood samples were used for blood counts and to isolate peripheral blood monocyte cells (PBMC).

### Ethics approval

In vivo experiments were carried out according to the guidance and regulation of the French Ministry of Higher Education and Research (MESR) with an appropriate staff, good animal practices and project authorizations (Protocol Number APAFIS#4422-201603081530483 v5). As part of this process, the experimental protocol was examined and approved by the appropiate local ethics committee, CREEA VdL (“Comité d’Éthique pour l’Expérimentation Animale Val de Loire”).

### Absolute lymphocyte counts by flow cytometry

Venous occipital sinus blood samples were collected into a commercial EDTA coated blood collection device (Monovette^®^, Sarstedt, Nümbrecht, Germany). Blood samples were kept at room temperature and processed within 4 h after blood collection. Lymphocyte (B, CD4^+^T, CD8^+^T) and thrombocyte counts were performed based on the Seliger method [40]. Five different mAb allowed the recognition of distinct sub-populations: the leukocytes (16-6-APC), CD8^+^T-cells (CT8-FITC), CD4^+^T-cells (CT4-RPE), B-cells (AV20-PerCP-Cy5.5) and thrombocytes (K1-RPE). Herein, monocytes were not counted. The thrombocyte count was used as a sample quality check to ensure that no blood clotting occurred, modifying leukocyte counts. Samples with blood clotting were discarded from the results. Measurements were performed on a BD LSR Fortessa (Becton–Dickinson, Heidelberg, Germany) with four fluorimetric lasers (blue laser-488 nm, yellow green laser-561 nm, red laser-640 nm, violet laser-405 nm) in BD Trucount^®^ absolute counting tubes (#340334, BD Biosciences). Acquisition was performed with BD FACSDiva software and data were analyzed with FlowJo software (Tree Star Inc., OR, USA).

### Histology and mitotic index count

Lymphoid tissue samples were fixed with 4% neutral buffered formalin, embedded in paraffin wax, transversally cut into 5 µm thick sections and stained using a routine hematoxylin–eosin–safranin (HES) staining method. All samples were observed by a skilled pathologist in a double-blind reading manner and lesions were systematically recorded.

In the bursa and thymus, mitotic nuclei were numbered in 10 randomly selected high-magnification fields. Mitotic index was then expressed as a mean number of mitoses per field. Intra-observer agreement was tested by reproducing this measure five times on the same sample and coefficient of reproducibility was above 90% for both tissues.

### DNA extraction from PBMC or lymphoid organs

Venous occipital sinus blood samples were collected into tubes containing 3% sodium citrate. PBMC were isolated using density gradient centrifugation on MSL (Eurobio, France). PBMC were counted using KOVA Glasstic Slide 10 (Hycor Biomedical Inc.). DNA extraction was performed on 10^7^ PBMC using the DNA Purification “Blood or Body Fluids Spin Protocol” of the QIAamp DNA mini Kit (Qiagen, Hilden, Germany). Incubation time with proteinase K at 56 °C was extended from 10 min to 2 h to increase DNA yield.

Approximately 25 mg of each organ (thymus, bursa or spleen) were disrupted mechanically with a micropotter in a 1.5 mL tube in 80 µL of PBS according to Qiagen recommendations. DNA extraction was performed using the DNA purification “tissues protocol” of the QIAamp DNA mini Kit (Qiagen). Tissues were incubated overnight at 56 °C with proteinase K to ensure efficient lysis. DNA concentrations were measured with a NanoDrop spectrophotometer.

### Quantification of MDV genome copy number by real-time PCR

Real-time PCR was performed using TaqMan technology, as previously described [41, 42]. Both iNos and the ICP4 probes were tagged with FAM-BHQ1. All qPCR were performed independently with 250 ng DNA, 10 pmol of each gene-specific primer, 5 pmol of the gene-specific probe in a 20 μL volume on a CFX96™ Real Time C1000 Touch™ Thermal Cycler (BioRad, Marnes-la-Coquette, France). The results were analyzed using the CFX Manager software (version 3.1) (BioRad). For each sample, viral DNA (based on ICP4 gene) and cellular DNA (based on iNos) were quantified independently in triplicates. The positive cut-off points corresponded to 23 copies for ICP4 and 57 copies for iNos. For each sample, the number of MDV genome copies was calculated per 10^6^ cells.

### Lytic MDV antigen detection on cryosections by fluorescence microscopy

Detection of lytic MDV antigens was performed on 7 µm cryosections of bursa and thymus. Sections were fixed in 4% paraformaldehyde (PFA), permeabilized and blocked in a PBS solution containing 10% BSA and 0.1% Triton. Cryosections were next incubated overnight at 4 °C with an antibody cocktail mix directed against three MDV antigens (ICP4, VP22 and gB) diluted (1:1000 each) in a PBS solution containing 1% BSA, 0.1% Triton. Replicate sections were treated under identical conditions with the diluent only, to serve as negative controls. After PBS washes, cryosections were then incubated 45 min with a secondary antibody, GAM-Alexa 488 (IgG) (1:2000) for the bursa and GAM-Alexa 488 F(ab’)^2^ (1:500) for the thymus. After PBS washes, cryosections were stained with Hoechst-33342 (1:2000) for 1 min and mounted with Mowiol^®^ 4–88 (Calbiochem). All cryosections were observed under an Axiovert 200 M inverted epi-fluorescence microscope equipped with the ApoTome system (Zeiss). Images were captured with a CCD Axiocam MRm camera (Zeiss) using the Axiovision software (Zeiss).

### TUNEL assay on cryosections and signal quantification

The detection of apoptotic cells in lymphoid organs were performed by TUNEL assay on 7 µm cryosections using the In Situ Cell Death Detection Kit, Fluorescein (#11684817910, Roche), according to the manufacturer’s protocol. Briefly, PFA-fixed cryosections were permeabilized with 0.1% Triton X-100 in 0.1% sodium citrate solution on ice for 2 min. TUNEL enzyme and label solution were mixed and applied on tissues, which were incubated in a humidified chamber for 1 h at 37 °C. Replicate sections were treated with the label solution only as negative controls. Next, cryosections were stained with Hoechst-33342 (1:2000) for 1 min, washed rapidly, mounted with Mowiol^®^ 4–88 and observed on Zeiss inverted fluorescent microscope (see above).

The quantification of a TUNEL positive signal was performed on high magnification digital images using FIJI^®^ software. First, we set a threshold of fluorescence from negative control images by using the “threshold function”; a different threshold was determined for thymus and bursa. The appropriate threshold was next applied on each image in order to keep all pixels that are above this threshold and considered as positive. Subsequently, all the positive pixels were counted with the “area measurement tool” on three different images for each sample. Lastly for each sample, the mean area was calculated from the three images.

### Double-labeling of MDV-infected cells and TUNEL positive cells on cryosections by fluorescence microscopy

Expression of viral antigens was detected in the thymus and bursa harvested from the MDV-infected group. Cryosections of 7 µm from each organ were first stained with primary antibodies directed against various MDV antigens following the protocol described above. The antigens detected were the following: VP22, ICP4 and gB at 6 dpi; VP22, ICP4, gB and Meq at 10 dpi; Meq alone at 14 dpi. The secondary antibodies used were coupled to AlexaFluor 488 and similar to the ones described above. Cryosections were next labeled by TUNEL with the In Situ Cell Death Detection Kit, TMR red (#12156792910, Roche) according to the manufacturer’s protocol. Finally, tissues were stained with Hoechst-33342 (1:2000) for 1 min, mounted with Mowiol^®^ 4–88 (Calbiochem) and observed on a Zeiss inverted fluorescent microscope (see above).

### Detection of PCNA (proliferating cell nuclear antigen) on paraffin sections by immunochemistry and signal quantification

Bursa and thymus tissue samples were fixed in 4% neutral buffered formalin and embedded in paraffin wax. Cells in division (S-phase) were detected using immunohistochemical staining of PCNA. Staining was performed on deparaffinized sections using a polyclonal rabbit anti-human PCNA antibody revealed with the EnVision HRP anti-rabbit Kit according to the manufacturer’s protocol (Dako, Les Ulis, France). Replicate sections were treated under identical conditions with the diluent only, to serve as negative controls. More precisely, after deparaffinization, the sections were treated with 0.1% trypsin for 10 min at 37 °C and placed in a 0.01 M citrate buffer (pH 6.0) for 20 min at 98 °C. The sections were allowed to cool and were covered with the Peroxidase Block solution of the EnVision Kit to block endogenous peroxidase activity. After incubating tissue specimens with the anti-PCNA (1:1000) for 30 min, immunoreaction complexes were detected by incubation for 30 min with the Peroxydase Labelled Polymer (ready to use). Positive signal was visualized with diaminobenzidine (DAB + substrate + chromogen solution from EnVision Kit). Sections were then counterstained with Gills’s hematoxylin and mounted in Eukitt (#03989, Biochemika). Sections were next observed on a NIKON microscope equipped with a Nikon DS-Ri color-camera. Color images were captured at different magnifications for subsequent quantitative analysis.

To determine the number of PCNA-positive cells relative to the total number of cells, we used the FIJI^®^ software. For each sample, on two different color images at high magnification, we defined three regions of interest (ROI) of 300 × 300 pixels each. Next, we used the “Immunohistochemistry (IHC) Image Analysis Toolbox” plugin in order to count automatically the number of PCNA-positive nuclei that appeared in brown on each ROI. The total cell number on the same ROI was then numerated manually using the “multipoint tool” of the software. Lastly, the percentage of PCNA-positive cells per sample was calculated over a total of at least 250 cells.

### Statistical analysis

Data were analyzed using Prism 6 software (GraphPad, La Jolla, California, USA). Differences between the groups were established either by the Kruskal–Wallis test and/or a Mann–Whitney test. A *p* value < 0.05 defined the level of statistical significance (*0.01 < *p* < 0.05; **0.001 < *p* < 0.01; ***0.0001 < *p* < 0.001; ****0.00001 < *p* < 0.0001).

## Results

### MDV induces a severe atrophy of the thymus and of the bursa at early time points post-infection

**Figure 1:**
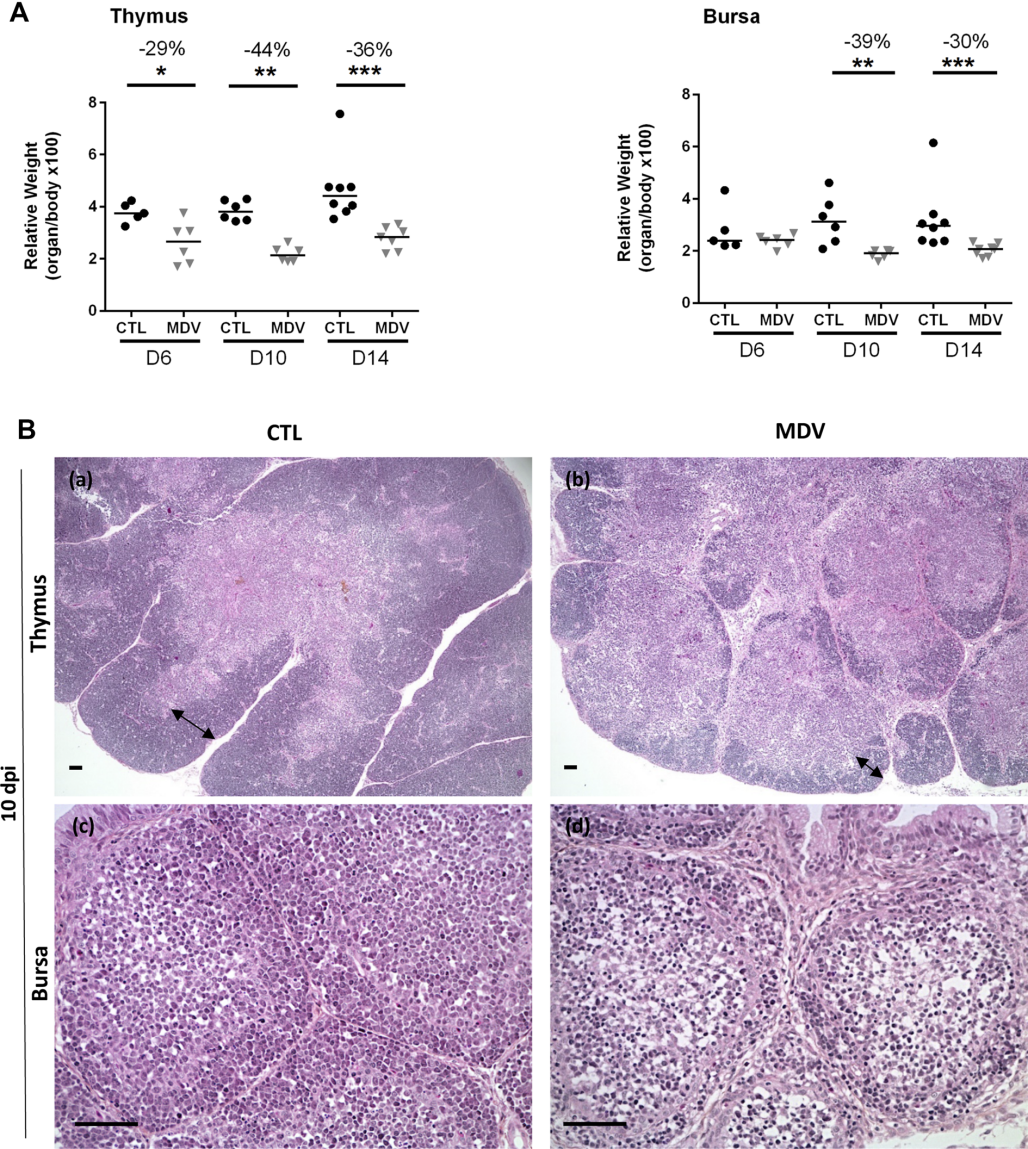
**Thymus and bursa atrophy induced by the vvMDV RB-1B strain in White Leghorn B19/B19 chicks. A** Organ weight/body weight ratios in CTL and MDV-infected chicks at 6, 10 and 14 dpi. The median is represented as a black line. Thymus and bursa relative weights were reduced significantly from 6 to 10 dpi respectively, in the MDV-infected group compared to the CTL group. **B** Histological structure of the thymus and the bursa at 10 dpi. Formalin-fixed tissues embedded in paraffin were sectioned and stained with HES. For the thymus, the cortex thickness (double-end arrow) was highly reduced in MDV-infected chicks (b) compared to controls (a). For the bursa, a massive cell depletion was observed in the medulla of the follicles in MDV-infected chicks (d) compared to the controls (c). Bar, 50 µm

To verify the intensity and kinetics of MDV-induced thymus and bursa atrophy in our in vivo experimental model, we injected vvRB-1B MDV-infected CESC (MDV group) or uninfected CESC (CTL group) intramuscularly in 2-day-old White Leghorn B19/B19 chicks. Five to six birds from each group were euthanized at 6 and 10 dpi and eight birds at 14 dpi and the relative lymphoid organ weight was calculated. We detected a significant atrophy of the thymus and of the bursa in the MDV group compared to the control group at 6 and 10 dpi respectively (29% in the thymus at 6 dpi; 44% for the thymus and 39% for the bursa at 10 dpi) (Figure 1A). The organ atrophy was persistent at 14 dpi with a lower intensity (36% in the thymus and 30% in the bursa) and compatible with a beginning of recovery of the organs (Figure 1A). All birds showed a splenomegaly from day 6 pi (+ 94% at 6 dpi, + 169% at 10 dpi and + 132% at 14 dpi) (Additional file 2). Body weight was not significantly reduced in MDV-infected birds compared to controls at all time points (data not shown). Major changes in the tissue structure were observed on sections stained with HES from day 6 pi, for all MDV-infected chicks (not shown). At 10 dpi, the thymic cortex thickness was highly reduced, with almost no cortex detectable in most lobules. The bursa displayed a massive lymphocytic depletion, with notably very few cells remaining in the medulla, accompanied with a global reduction in bursa follicle size (Figure 1B). At 14 dpi, the differences in the thymus were less extended. A slight lymphocyte depletion persisted in the thymic cortex with numerous tangible body macrophages in MDV-infected birds compared to controls (not shown).

**Table 1:** **Number of birds positive for MDV DNA and expression of MDV lytic antigens in different tissues**

Day pi	Number of birds positive for MDV genome by qPCR				Number of birds positive for MDV lytic antigens	
	Thymus	Bursa	PBMCs	Spleen	Thymus	Bursa
6	6/6	6/6	6/6	6/6	6/6	6/6
10	6/6	6/6	6/6	6/6	2/6	3/6
14	8/8	8/8	8/8	8/8	0/8	2/8

### MDV genome and lytic antigen expression were detected in the thymus and bursa before atrophy occurred

**Figure 2:**
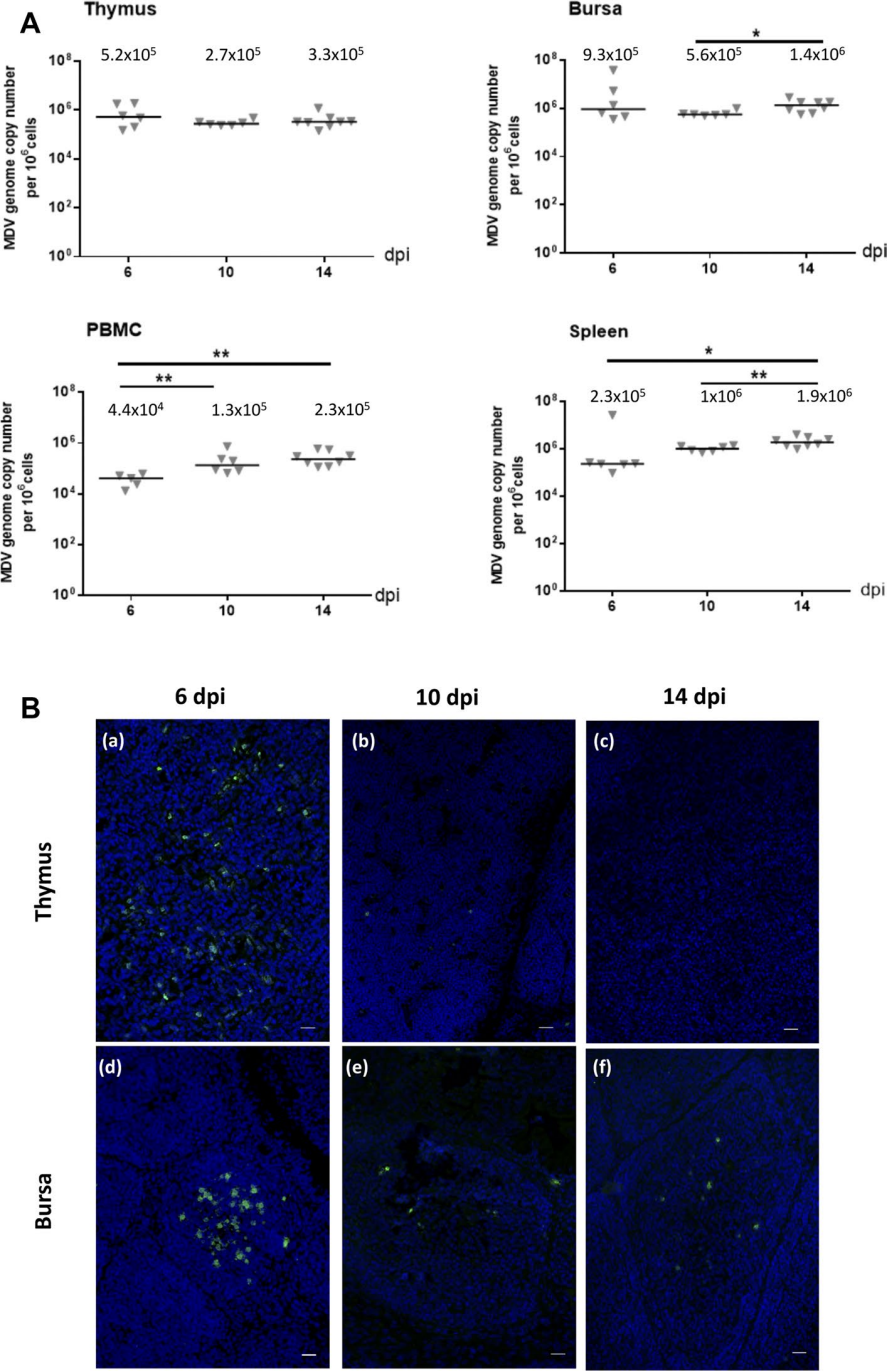
**MDV replication in lymphoid organs of infected-chicks at early time points. A** MDV DNA loads in lymphoid organs of MDV-infected chicks. The MDV genome copy number per million cells was quantified at 6, 10 and 14 dpi using a Taqman real-time qPCR in thymus, bursa, PBMC and spleen. The viral loads were already high since 6 dpi in thymus, bursa, PBMC and spleen of all infected chicks and no significant or little further increase were detected at later time points. The median is represented as a black line. **B** Expression of lytic viral antigens in the thymus and the bursa. Cryosections of both thymus (a–c) and bursa (d–f) at 6, 10 and 14 dpi were stained with a cocktail of three mAb directed against MDV lytic antigens (VP22, ICP4, gB) (Green). Nuclei were stained with Hoescht 33342 dye (blue). MDV lytic antigens were detected in all birds in both primary lymphoid organs at 6 dpi; in 2/6 chicks for the thymus and 3/6 chicks for the bursa at 10 dpi and in 2/8 chicks for the bursa at 14 dpi (no detection in thymus at 14 dpi). In the bursa, MDV-infected cells in lytic cycle were predominantly located in the medulla at 6 dpi. Bar, 20 µm

To investigate MDV replication in the thymus and bursa of MDV-infected chicks, the MDV genome was quantified in these organs as well as in PBMC and spleen by real-time quantitative PCR using the Taqman method. Organs and PBMC from the control group were verified to be MDV-free. The MDV genome was detected in the four tissues of all infected birds at 6 dpi and after (Table 1). At that time, the median viral load was 5.2 × 10^5^ copy number per million cells in the thymus, 9.3 × 10^5^ in the bursa, 4.4 × 10^4^ in PBMC and 2.3 × 10^5^ in the spleen (Figure 2A). The MDV load remained globally constant in the thymus and bursa until 14 dpi and showed a slight increase over time in the PBMC and the spleen. At 14 dpi, MDV load reached 2.3 × 10^5^ in the PBMC and 1.9 × 10^6^ in the spleen. This result indicates that high MDV viral loads are present in primary lymphoid organs, before the atrophy of the bursa and before the peak of thymus atrophy.

As the detection of MDV DNA is not necessarily associated with a cytolytic productive infection, we next studied the expression of MDV lytic antigens in the thymus and bursa over time in MDV-infected and control birds. For that, cryosections of these organs were stained with antibodies against three highly expressed lytic antigens (ICP4, VP22 and gB) (Figure 2B). All birds from the control group were exempt of positive signals. At 6 dpi, all chicks from the MDV-infected group were positive for MDV lytic antigens in the thymus and bursa, indicating that MDV already actively replicated in these organs at that time (Table 1). At 10 dpi, MDV lytic antigens were detected in the thymus of 2/6 chicks and in the bursa of 3/6 chicks from the MDV-infected group (Table 1). At 14 dpi, no thymus sample was found positive for lytic antigen expression, but a positive signal was observed in the bursa of 2/8 chicks from the MDV-infected group, indicating that the lytic antigen expression lasted longer in the bursa than in the thymus of a few birds (Table 1). This result indicates that MDV replication stopped earlier in the thymus than in the bursa, possibly by getting into latency.

In the thymus, at 6 dpi, numerous MDV-positive cells were detected, either isolated or in clusters (Figure 2B, panel a). At 10 dpi, rare sparse MDV-positive cells were visible in the thymus of the birds scored positive. Considering the nuclear density and the cortex atrophy, infected cells appeared mostly located in the medulla.

At 6 dpi, MDV-positive cells in the bursa were usually clustered and located predominantly in the medulla (Figure 2B, panel d), even if a few isolated cells were sometimes detectable in the cortex or in the medullo-cortical region. One infected follicle was either isolated or in the vicinity of one or several other infected follicles. At 10 dpi, MDV-positive cells were rarely observed. At 14 dpi, when MDV-positive cells were visible, they were also rare and sparse (Figure 2B, panel f), except once when one large plaque was observed (not shown).

### MDV increases apoptosis in the thymus and also in the bursa

**Figure 3:**
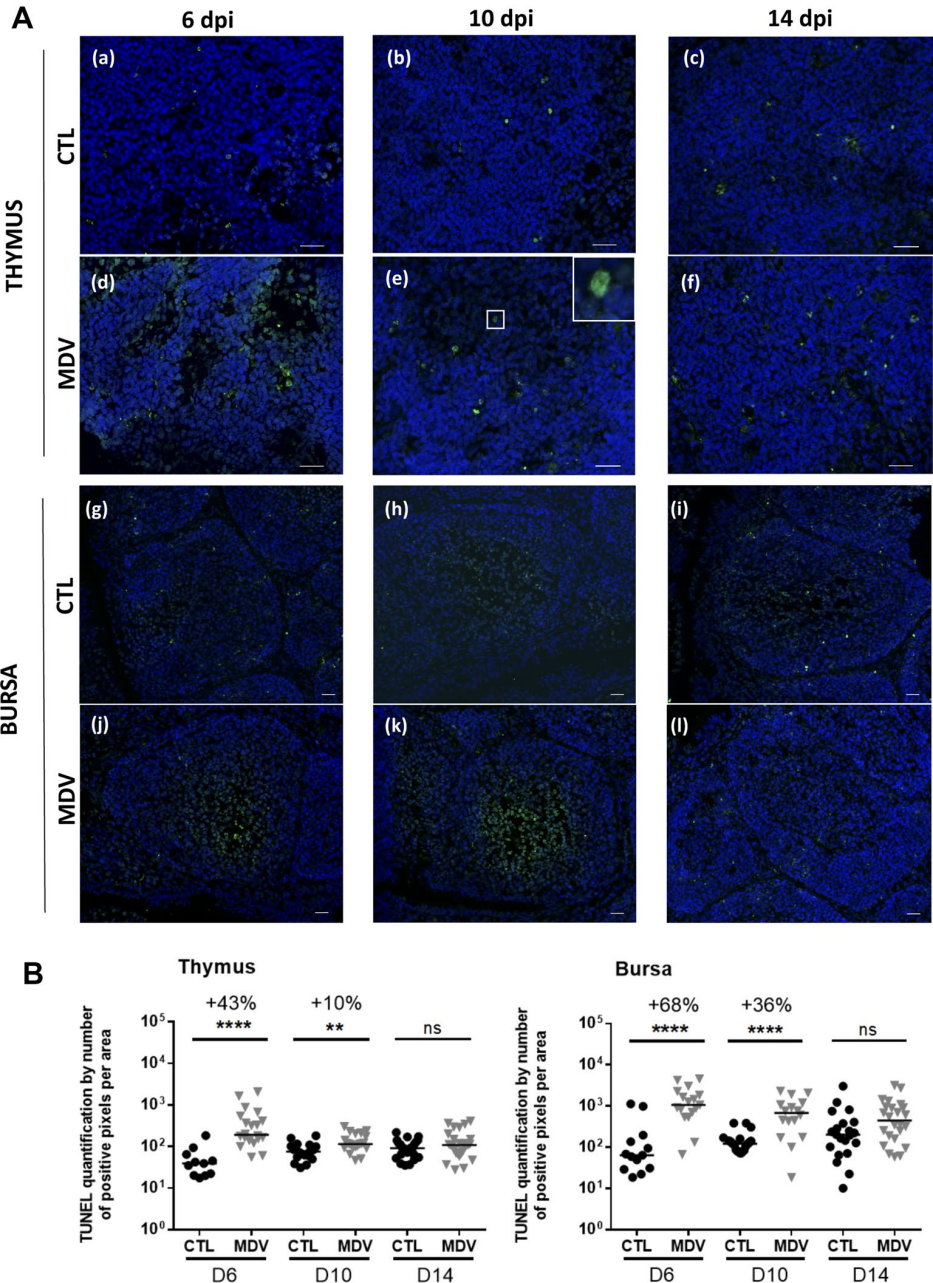
**MDV increases apoptosis in the thymus and the bursa at 6 and 10 dpi. A** Cryosections of thymus (a–f) and bursa (g–l) harvested on CTL and MDV-infected birds at 6, 10 and 14 dpi were stained by TUNEL assay (green) and the nuclei counterstained with Hoescht 33342 dye (blue). TUNEL positive cells presented green nuclei, as shown enlarged (× 3) in the corner panel (e). Apoptosis was increased in the thymus of MDV-infected chicks compared to controls at 6 dpi, especially in the medulla. For the bursa, apoptosis was increased at 6 and 10 dpi, especially in the medulla. **B** Quantification of the green fluorescent signal on cryosections stained by TUNEL assay. This measurement was performed by area determination with FIJI software on three pictures per organ/chick/time. The median is represented with a black line. Apoptosis was highly enhanced at 6 dpi in the thymus and the bursa and to a lesser extend at 10 dpi compared to CTL. No differences in apoptosis were observed between infected- and CTL chicks at 14 dpi

In order to examine the influence of MDV infection on apoptosis in the thymus and in the bursa, we performed an in situ TUNEL assay on cryosections of both organs from control and MDV-infected chicks at 6, 10 and 14 dpi. Physiological apoptosis is known to occur in primary lymphoid organs representing the physiological turnover of lymphocytes during their maturation. In MDV-infected chicks, apoptosis signals were increased predominantly at the cortico-medullary junction of the thymus and in the medulla of the bursa at 6 and 10 dpi (Figure 3A). As expected, the TUNEL signal was localized in the nucleus (Figure 3A, panel e). After subtraction of the background signal, the global TUNEL-FITC signal for thymus and bursa was quantified at each time point on each cryosection. At 6 dpi, a significant increase of the apoptotic signal was observed in the thymus (+43%) and bursa (+68%) of MDV-infected chicks compared to the controls. At 10 dpi, the augmentation of the apoptotic signal was still visible to a lower extent (+10% in the thymus and +36% in the bursa). At 14 dpi, no significant difference was detected between the two groups for both organs (Figure 3B).

### MDV increases apoptosis mostly in uninfected cells in the bursa in contrast to the thymus

**Figure 4:**
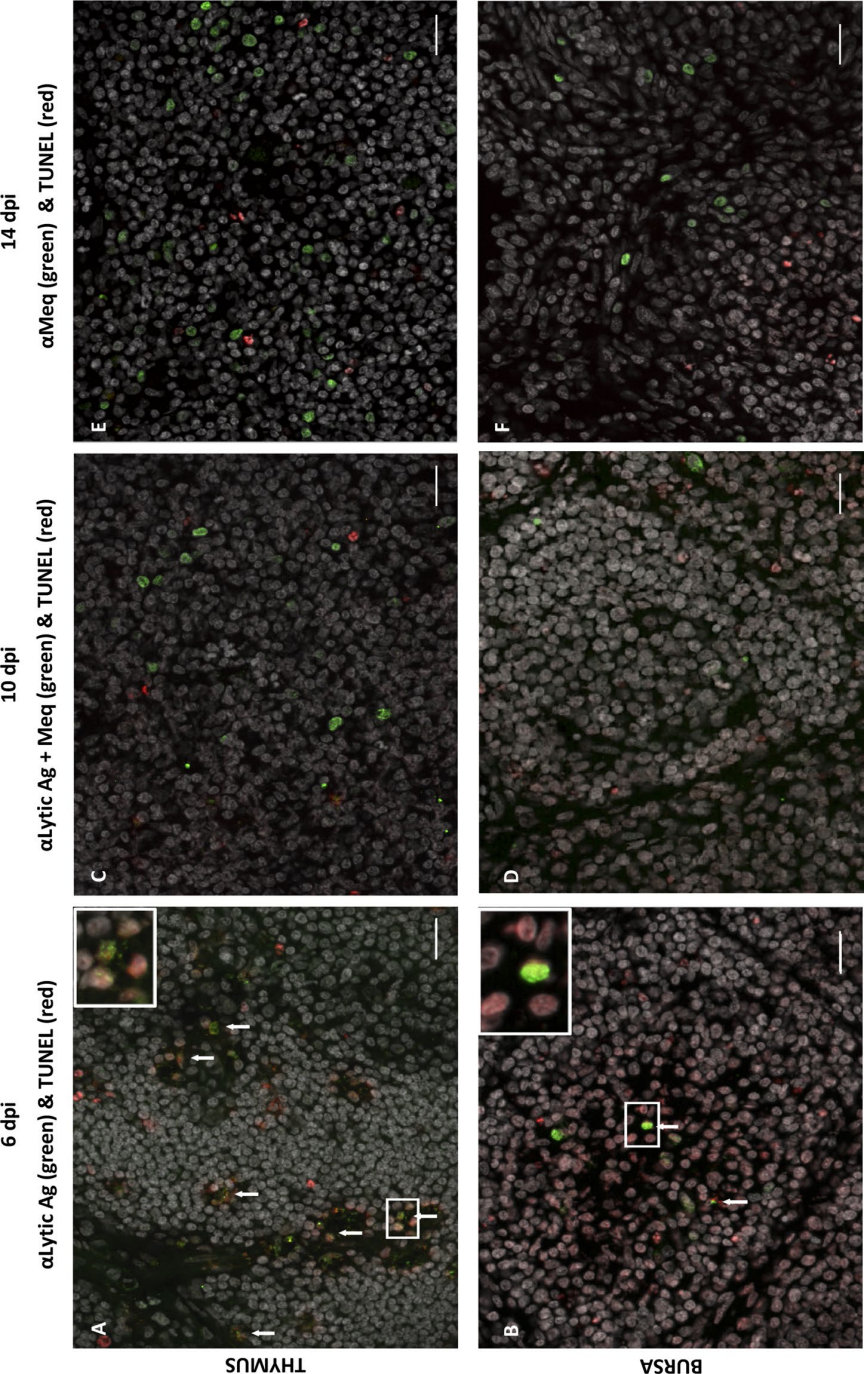
**Cells expressing MDV antigens are apoptotic in the thymus and rarely in the bursa.** Cryosections of thymus (**A**, **C**, **E**) and bursa (**B**, **D**, **F**) were stained for MDV antigens (lytic: VP22, ICP4, gB and/or latent: Meq) (green) and apoptosis was detected by TUNEL assay (red). DNA content of the cells were stained with Hoescht 33342 dye (white). MDV antigens positive cells and apoptotic cells were detected in both organs at 6 dpi (**A**, **B**), 10 dpi (**C**, **D**) and 14 dpi (**E**, **F**). At 6 dpi, in the thymus, the majority of the cells expressing the lytic MDV-antigens were apoptotic, in contrast to the bursa. At 6 dpi, in the bursa, only rare double positive cells were detected (as shown in panel B, in the enlarged frame). In both organs, at 10 and 14 dpi, no co-localization between MDV antigens (including Meq) and TUNEL signal was observed. Bar, 20 µm

To identify whether apoptosis occurred only in MDV-infected cells or not, a double staining was performed to visualize both the TUNEL signal and MDV antigen expression. Herein, we used three mixes of antibodies detecting lytic antigens and/or the Meq protein depending on the time point. Note that Meq is predominantly expressed in latently infected cells, but also during the lytic cycle [43]. In the thymus, we observed a partial co-localization of MDV antigens and TUNEL signals at 6 dpi, indicating that some cells expressing MDV lytic antigens were apoptotic (Figure 4A). Such co-localization was not visible anymore at 10 and 14 dpi (Figures 4C and E). In contrast, in the bursa at 6 dpi, only rare cells positive for MDV lytic antigens were apoptotic (Figure 4B). In addition, apoptotic cells were often localized nearby cells expressing MDV lytic antigens in the medullary region of the bursa, but not only. At 14 dpi, the Meq protein was still found to be expressed in the thymus and the bursa of all birds with no co-localization between Meq and TUNEL signals (Figures 4E and F). In conclusion, MDV triggers apoptosis in the thymus and the bursa, from 6 to 10 dpi, along with MDV lytic antigen expression. In addition, if numerous cells undergoing apoptosis in the thymus were in lytic cycle, this is not the case in the bursa in which most apoptotic cells were uninfected.

### MDV infection is associated with an inhibition of cell proliferation from 6 to 14 dpi in the bursa, but not in the thymus

**Figure 5:**
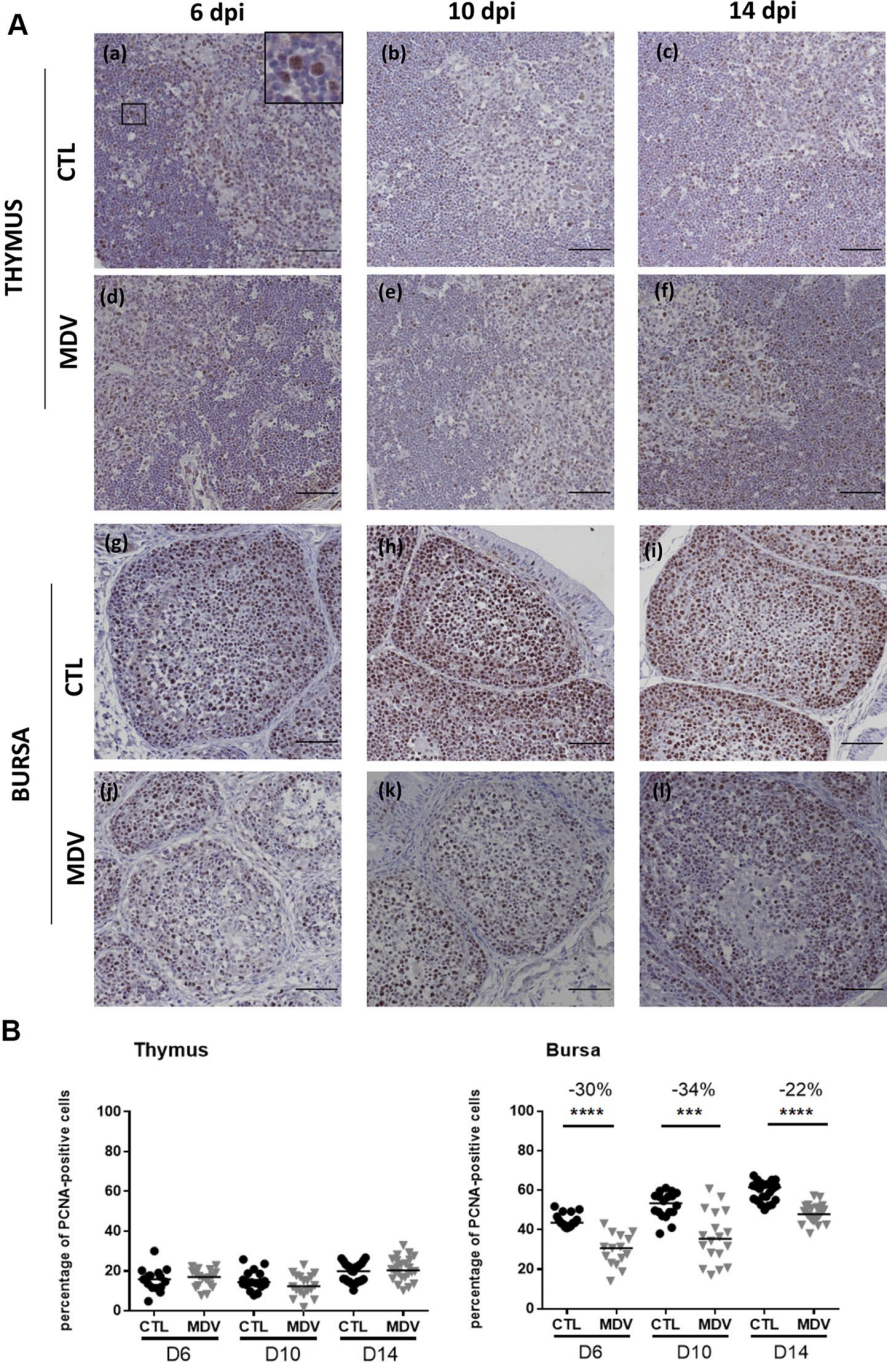
**MDV inhibits cell proliferation in the bursa from 6 to 14 dpi but not in the thymus. A** Proliferating cells were detected on both thymus (a–f) and bursa (g–l) of CTL and MDV-infected chicks at 6, 10 and 14 dpi by immunochemistry. For that, paraffin embedded sections were stained with a rabbit anti-cell nuclear antigen antibody (PCNA) revealed with EnVision anti-rabbit HRP kit after 3′diaminobenzidine (DAB) treatment (brown nuclei, as shown enlarged (×4) in the corner panel (a). The tissues were counterstained with Gill’s hematoxylin and the PCNA-negative nuclei appeared in blue. In the thymus, the number of PCNA-positive cells appeared to be equivalent in both groups of chicks. In the bursa, the total number of PCNA-positive cells was highly reduced in the infected group compared to the control group. PCNA-positive cells were mostly present in the bursal cortex of the control group. Bar, 50 µm. **B** Percentage of PCNA-positive cells in the bursa and the thymus at 6, 10 and 14 dpi. The median is represented with a black line. A significant reduction in the number of PCNA-positive cells was observed at each time point in the bursa, and not in the thymus

We previously reported that MDV infection of fibroblasts in vitro delays cell cycle progression [44]. Due to the low number of infected and apoptotic cells compared to the extent of organ atrophy, we examined whether MDV infection does impair cell proliferation. For that, paraffin sections of both thymus and bursa at 6, 10 and 14 dpi were immuno-stained for the proliferating cell nuclear antigen (PCNA). In the thymus, numerous PCNA-positive cells were observed in both the thymus cortex and medulla of the control birds (Figure 5A). The number of PCNA-positive cells was higher in the bursa than in the thymus of control birds. In the bursa, the density of PCNA-positive cells was higher in the cortex than in the medulla. Moreover, between days 6 and 14 (corresponding to 7 and 15-day-old birds), the bursal cortex became thicker with an increase in cell density, which is in accordance with the development of this region after hatching. In the thymus of MDV-infected chicks, the number of PCNA-positive cells was similar to the one in control birds, at all time points and confirmed by quantification (Figure 5B). In contrast, in the bursa, the number of PCNA-positive cells per sample in MDV-infected chicks was significantly reduced at all time points (−30% at 6 dpi, −34% at 10 dpi and −22% at 14 dpi), in both cortex and medulla compared to control birds (Figure 5B).

The PCNA antigen is expressed only during the S-phase in the cell cycle. Therefore, in order to confirm that the reduction in PCNA-positive cells was associated with a decrease in cell division, we next determined the mitotic index in the thymus and in the bursa of 5–6 animals at every time point. The number of mitotic cells was not significantly reduced in the thymus of the MDV-infected group compared to the control group at any time point (Table 2). In contrast, a significant reduction of the number of mitotic cells in the bursa was observed in the MDV-infected group in comparison to the control group, at 6 and 10 dpi, but not at 14 dpi.

**Table 2:** **Mitotic index determination over time in thymus and bursa sections**

Day pi	Group	Thymus	Bursa	
6	CTL	7.9 ± 3.8	21.8 ± 5.9	*n* = 5
	MDV	5.3 ± 2.2	9.9 ± 4.5^a^	*n* = 5
10	CTL	8.6 ± 6.2	16.7 ± 4.7	*n* = 6
	MDV	5.7 ± 2.8	9.2 ± 3.2^a^	*n* = 6
14	CTL	11.1 ± 4.9	21.8 ± 6.3	*n* = 6
	MDV	7.6 ± 3.4	15.7 ± 4.2	*n* = 6

^a^Mitotic index was reduced significantly in MDV-infected bursa at 6 and 10 dpi compared to controls, but not in MDV-infected thymus

### MDV infection induces a drastic decrease of the number of B-lymphocytes in the blood during the first 2 weeks of infection

**Figure 6:**
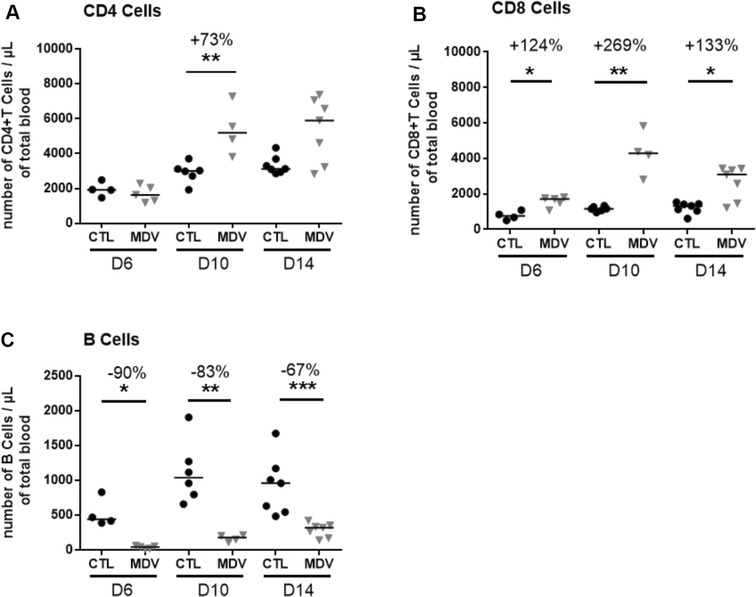
**MDV infection induces a significant decrease in B-lymphocytes blood count at early time points.** EDTA-blood of each chick was stained with anti-CD4-RPE, anti-CD8-FITC, anti-Bu1-PerCP-Cy5.5, anti-K1-RPE, and anti-CD45-APC in a no-lyse no-wash single-step procedure and analyzed by flow cytometry. Absolute number of T-CD4^+^ (**A**), T-CD8^+^ (**B**) and B lymphocytes (**C**) per µL of blood is given for most birds at each time point (6, 10 and 14 dpi). A few birds are missing because of partial blood clotting detected by cytometry. The median is represented with a black line. A significant increase was observed for T CD4^+^ cells at 10 dpi and for T CD8^+^ cells at all time points with a peak at day 10. Inversely, a significant decrease between 90 and 67% was observed for B-cells at 6, 10 and 14 dpi

Because the atrophy of the primary lymphoid organs is related to a severe lymphocyte depletion in these organs, we were wondering if this atrophy could also be associated with a diminution of the number of lymphocytes circulating in the blood. To test this, we performed lymphocyte counts in the total blood by flow cytometry with an adapted assay developed for the purpose of this study, at 6, 10 and 14 dpi (Figure 6). As expected, in the control group, each population of lymphocytes (CD4^+^T, CD8^+^T and B) gradually increased over time reflecting the development of cellular immunity and the lymphocyte release from primary lymphoid organs. In addition, an individual heterogeneity was observed in this group, especially in the number of B-cells which ranged from 388 to 830 in 7-day-old, 659 to 1904 in 11-day-old, 484 to 1673 in 15-day-old B19 chicks. In the MDV-infected group, the number of CD4^+^ and CD8^+^T increased more than in the controls, especially CD8^+^T-cells. Indeed, the CD8^+^T-cell number was significantly higher from 6 dpi on (+124%) with a peak at 10 dpi (+269%). For the CD4^+^T-cells, a significant increase was observed only at 10 dpi (+73%). In both cases, the increase in T-cells might reflect the host cell response against the viral infection. In contrast, the number of B-lymphocytes increased very slowly in the MDV-infected group compared to the control group. In the infected group, B-cell number ranged from 14 to 62 at 6 dpi, from 109 to 213 at 10 dpi and from 169 to 423 at 14 dpi. Therefore, the number of B-lymphocytes was drastically reduced in the MDV-infected group compared to the control group (age-matched) at each time point (−90% at 6 dpi, −83% at 10 dpi and −67% at 14 dpi).

## Discussion

Marek’s disease alphaherpesvirus is well known to induce an atrophy of the thymus and the bursa during early infection. Herein, with the vvRB-1B strain in White Leghorn B19 chicks, thymus and bursa atrophy was observed from 6 and 10 dpi, respectively, peaking at 10 dpi with about 40% of atrophy. Earlier studies reported that the degree of thymus and bursa atrophy depends mostly on the virulence of the MDV strain [16, 45]. Therefore, in order to validate our in vivo experimental conditions, we compared our results with those previously obtained with the RB-1B strain [16, 46–48]. The level of thymus and bursa atrophy was of the same intensity at 8 and 14 dpi as reported before [16]. Although we recorded microscopic lesions at 6 dpi in both organs, we did not observe bursa atrophy at this time point. The bursa atrophy appears a little delayed in our study, since this atrophy is usually visible from 7 dpi [47, 48]. This temporal difference might be attributable to non-viral parameters known to slightly modulate primary lymphoid organ atrophy, such as chicken line and age [3, 16, 49]. In particular, we cannot totally discard a 1/2-day-delay in MDV replication in the bursa in our experimental conditions. Indeed, although viral loads (as determined by MDV genome copy number and detection of MDV lytic antigens) were elevated at 6 dpi in the bursa and thymus, we did not examine the viral loads and expression of MDV lytic antigens at earlier time points. It is important to remember here that this phenotype was obtained in highly susceptible SFP birds. In the field, most of the chicks have maternal antibodies due to hen vaccination with HVT, SB-1 and/or Rispens and are genetically less susceptible. Therefore, they might present a different dynamic of virus infection in the lymphoid organs. Previous studies showed that HVT maternal antibodies failed to protect chicks against MDV induced early atrophy of the bursa and the thymus as well as against MDV replication in these organs. On the contrary, chicks carrying MDV maternal antibodies due to previous infection in the flock are quite well protected [17, 50].

Our results show a rise in the number of apoptotic cells in the thymus at 6 dpi, whereas no apoptosis was detected at 14 dpi. These observations were in accordance with previous observations reporting a transient increase of apoptosis in the thymus during early MDV-infection at 7 dpi [18, 51]. In addition, we demonstrate that the level of apoptosis is concomitant with the expression of MDV lytic antigens in the thymus and to its extent in term of cell number. Indeed, at 10 dpi, a low increase of apoptotic cells was still detectable (+ 10%) with a low number of cells expressing lytic antigens. Moreover, apoptotic cells had totally disappeared at 14 dpi, when lytic antigens (VP5, ICP4, gB) became undetectable in this tissue, although numerous latently infected cells persisted as evidenced by Meq detection. This last observation was in accordance with the anti-apoptotic property of Meq [52]. We also clearly evidenced that the majority of cells expressing MDV lytic antigens at 6 dpi were apoptotic. Therefore, this analysis validates the general idea that thymic cells replicating MDV subsequently die of apoptosis and that this process is transient upon early MDV infection.

How MDV infection triggers apoptosis in thymic cells has not yet been investigated. We can speculate on various molecular mechanisms reported for other pathogens in lymphocytes. (i) A MDV lytic gene could directly trigger apoptosis (as observed with the apoptin and the gp120 encoded by CAV and HIV-1, respectively [53, 54]). The tegument protein VP22 encoded by MDV could be a good candidate since this protein arrests the cell cycle in S-phase and induces DNA damages after overexpression in proliferating avian cells [44]. (ii) An MDV gene could also trigger apoptosis indirectly by interfering with the thymocyte positive selection. In such hypotheses, the virion host shutoff (VHS) endoribonuclease appears as a candidate of choice because this enzyme is well known to inhibit cellular protein synthesis during productive infection by mediating the degradation of stable mRNA [55]. The pUL49.5 of MDV known to down-regulate MHC-I molecule is another possible viral candidate [56], for which the effect on thymocytes was never explored. (iii) The killing of infected cells by Natural Killer (NK) cells could also be an explanation of the death of infected cells by apoptosis, even though the presence of NK cells in the thymus during MDV cytolytic infection has not been shown yet.

Strikingly, in the thymus, we also often observed apoptotic cells that were not expressing MDV lytic antigens. As we used an antibody directed against ICP4, an immediate early MDV antigen, it is unlikely that these cells were in the very early steps of infection. Therefore, we conclude that MDV infection also promotes cell death of uninfected cells in the thymus. In this process, the direct contribution of viral factors or indirect contribution of the host response, as through soluble immune mediators (e.g. TNF or IFN-γ and nitric oxide) or glucocorticoids, might be studied.

One of the most important findings in our study is the cell perturbations induced by MDV infection in the bursa. This study provides the first evidence that two cellular processes occur in this organ between 6 and 14 dpi: an increase of cell death by apoptosis and an inhibition of cell proliferation. First, the increase of apoptotic cells was located in the medulla of bursal follicles, the region in which MDV replicates actively as detected by the expression of lytic antigens microscopically. This result was in accordance with the high lymphocyte depletion observed in the medulla compared to the cortex. Intriguingly, we show that most apoptotic cells detected in the bursa of infected chicks at 6 dpi were negative for MDV antigens, in contrast to what was observed in the thymus. This indicates that MDV infection of the bursa highly enhanced cell death by apoptosis of uninfected cells. An increased death by apoptosis of uninfected cells in the bursa after viral infection was already reported for IBDV in vivo [57]. However, in this case, IBDV apoptotic antigen-positive cells were also present. Finally, it is important to point out that in the bursa like in the thymus, apoptosis occurs simultaneously to productive infection, indicating that the two processes are temporally associated.

Secondly, we observed a decrease of cell proliferation in the cortex and medulla of the bursa. This observation was surprising because infected cells in the lytic cycle were predominantly detected in the medulla, as reported earlier [48, 58]. Furthermore, in contrast to apoptosis, the decrease of cell proliferation occurred concomitantly with MDV lytic antigen expression and persisted beyond. Therefore, MDV lytic infection seemed to trigger cell proliferation inhibition, but was not totally necessary for its persistence. Altogether, these results suggest the involvement of different molecular determinants in apoptosis and cell proliferation inhibition in the bursa. Moreover, the fact that uninfected cells are the major targets of apoptosis suggests that this process is mediated by the secretion of soluble factors inducing cell death or by the privation of soluble factors favoring cell survival. Lastly, it is important to note that the cortex develops after hatching by the migration of B-cells from the medulla to the stromal region under the basal membrane. In the juvenile bursa, the cortex is considered as the most active zone of the follicle for cell proliferation [59]. Interestingly, in MDV-infected chicks at hatching, although numerous bursal follicles were atrophied at 2 weeks pi, the cortex was present, indicating that MDV infection does not inhibit the development of this structure.

Another major point of interest of the present study is the drastic decrease of circulating blood B-cells that we observed in MDV-infected birds between 6 and 14 dpi, in newly hatched SFP B19 chicks compared to control age-matched birds. Such a blood B-cell reduction was previously observed in 2-week-old SPF Red Island chickens infected with the hypervirulent C12/130 strain or with the virulent HPRS-16 strain [60]. In these birds, the B-cell reduction started at 2–4 dpi depending on the virus. The lowest level of reduction was observed at 6 dpi, before increasing slowly at 10 dpi (despite being still 3.5 times lower than controls at that time). Considering our results and the report by Barrow et al., B-lymphopenia is induced by strains of different pathotypes in different SPF chicken lines of different ages. We propose that the B-cell number is a good indicator of early MDV-infection in SPF birds, associated with bursa atrophy. Interestingly, this B-lymphopenia was detected earlier than the bursa atrophy and lasted at least until 14 dpi, indicating that B-lymphopenia is an early and sustainable marker of the MDV infection. However, this biological parameter appears reliable if performed on several subjects and not on a single subject. Indeed, we showed herein a high individual heterogeneity in B-cell number per microliter in non-infected chicks of the same age, as was demonstrated earlier [40]. In addition, the absolute B-cell number median varies depending on the chicken line (unpublished data on White Leghorn B13) [40]). Therefore, to avoid misinterpretation of the results with this test, this method requires a prior establishment of blood B-cell count variation range in the chicken line of interest, according to age. Reduction of blood B-cells has also been demonstrated for IBDV in some studies, even if not all [61, 62]. This indicates that the decrease of blood B-cell number is a common feature of these two viral infections targeting the bursa and therefore cannot be considered as a pathognomonic marker of one of these two viral infections. Further investigations need to be done on chicks with maternal antibodies to HVT or MDV in order to see if blood B-cell number is also an interesting tool for diagnosis of lymphoid organ lesions induced by MDV in the field.

The origin of the decrease of blood B-cells induced by MDV is still not known. One can speculate (i) a cell-death of circulating B-cells infected by MDV and/or (ii) an inhibition of the B-cell emigration from the bursa to the periphery. Looking at B-cell numbers over time in MDV-infected birds, we noticed that the B-cell number in the blood was very low at 6 dpi and increased very slowly from 6 to 14 dpi, resulting in a global persistent decrease in blood B-cells from day 6 to day 14. As B-cell count was not examined at 3–4 dpi, we cannot totally exclude that a drop in B-cell number did not occur at that time. Therefore, even if we cannot exclude a contribution of the first scenario, the second one is likely predominant due to the blood B-cell curve, the massive lymphocyte depletion in the bursal follicles and the B-cell exit pathway from the bursa. Indeed, naive B-cells start to emigrate from the bursa around hatching and represent about 5% of the newly generated bursal B-cells, the others dying [59]. Our favorite hypothesis is that MDV infection of the bursa limits the emigration of B-cells into the periphery from the cortex and/or the medulla and that this effect lasts longer than the lytic infection of this organ, in accordance with the inhibition of cell proliferation still observed at 14 dpi in the bursa. In addition, we did not define if B-lymphopenia is due to the death or the inhibition of proliferation of specific B-cells or of any type of B-cells. This will be an important question to address in the future for a better understanding of the consequence of this transient lymphopenia.

Surprisingly, in RB-1B infected birds showing a thymus atrophy, we did not observe a decrease in T-cell number in the blood, but a T lymphocytosis of CD4^+^ and CD8^+^ cells. The increase of T CD8^+^ cells was massive and persistent at the three post-infection time points tested, whereas the increase of T CD4^+^ was lower and significant solely at 10 dpi (although 4/7 birds were still high at 14 dpi). Morimura et al. previously observed an increase of the percentage of T CD4^+^ at 10 and 15 dpi, and of the T CD8^+^ only at 15 dpi [39]. Barrow et al. who measured T-cells from 2 to 10 dpi, observed an increase of CD4 + and CD8 + at 8 and 10 dpi with HPRS16 and C12/130 [60]. Therefore, our results were globally concordant with these two previous studies.

Previous studies reported that early MDV-infection and even MD vaccination (Rispens or HVT/SB1) induce a transient immunosuppression, which is associated with a lower antibody response to various antigens (for e.g. *Mycoplasma synoviae* and BSA) [12, 14]. Interestingly, Mast and Goddeeris demonstrated that chicks immunized at 1 day of age with the BSA antigen, a thymus dependent antigen, developed a lower immunoglobulin response than chicks immunized at 7 or 14 days of age [63], probably due to an incomplete maturation of the peripheral B-cell compartment. Therefore, it is conceivable that the B-lymphopenia that we observed may delay the immuno-competence of chicks to develop antibodies against antigens, possibly by a retardation in B-cell entry into the spleen. We can therefore speculate that MDV infection might be more deleterious for inactivated vaccines than for live vaccines or immune complexes which remain in the body for a longer period of time and might still be present when B-cell number starts to re-increase.

In conclusion, until now, it was assumed that lymphocyte depletion in primary lymphoid organs was mostly due to the cell death of infected cells. Herein we demonstrate the death of uninfected cells, especially in the bursa. We also highlight the occurrence of another process in the bursa, the inhibition of cell proliferation and that B-cell depletion in the bursa is associated with a strong B-cell lymphopenia, which could become a good biological marker of early MDV cytolytic infection.

## Additional files

**Additional file 1.** **Characterization of the Lamba7 mouse monoclonal antibody anti-Meq.** The hybridoma supernatant was assayed against different cell substrates (A-E). The detection of meq antibody was performed with a goat anti-mouse conjugated to Alexa Fluor 488 (Green) and the nuclei stained with Hoechst 33342 (Blue). Labelled cells were observed by fluorescence microscopy. (A) Sf9 cells infected with a MEQ baculovirus. (B) ESCDL-1 cells transfected with pBK-MEQ-CMV. (C) MSB-1 and (D) DT40; for both cell lines, the actin filaments were stained with phalloidin conjugated to Alexa Fluor 594 (red). Meq is detected in the nucleus of all MSB-1 cells indicating that Lamba7 recognizes the Meq protein expressed during latency. As expected, Meq was not detected in DT40 cells. (E) ESCDL-1 cells infected with a recombinant rRB-1B pp38 mcherry. Meq was detected in the nuclei of cells in lytic cycle expressing pp38 mcherry. Bars are shown on each image.

**Additional file 2.** **Splenomegaly induced by the vvMDV RB-1B strain in SPF White Leghorn B19/B19 chicks.** Spleen weight/body weight ratios in CTL and MDV-infected chicks at 6, 10 and 14 dpi. The median is represented as a black line. The spleen relative weight was significantly increased from 6 dpi to 14 dpi, in the MDV-infected group compared to the CTL group.

## Abbreviations

bursa: bursa of Fabricius
CTL: control
dpi: day post-infection
HES: hematoxylin–eosin–safranin
mAb: monoclonal antibody
MDV: Marek’s disease virus
pi: post-infection
PBS: phosphate-buffered saline
PFA: paraformaldehyde
ROI: region of interest
SPF: specific pathogen-free
