# Supplementary figures and images for: Atrophy of primary lymphoid organs induced by Marek’s disease virus during early infection is associated with increased apoptosis, inhibition of cell proliferation and a severe B-lymphopenia
Source: Vet Res. 2018 Mar 27;49:31. doi: 10.1186/s13567-018-0526-x (PMC5870490; doi:10.1186/s13567-018-0526-x)

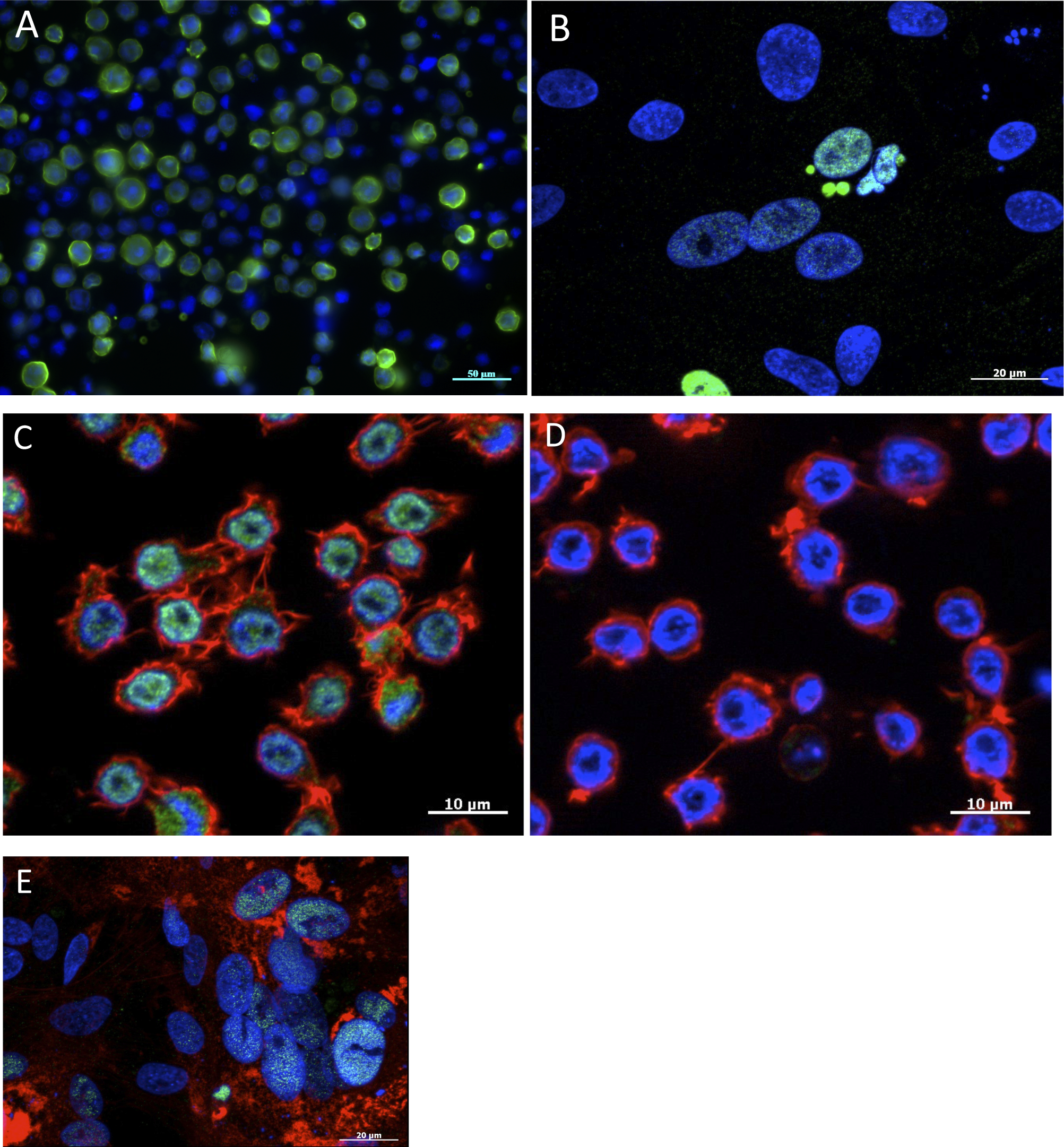

Supplement: Supplementary file 1 — Additional file 1. Characterization of the Lamba7 mouse monoclonal antibody anti-Meq. The hybridoma supernatant was assayed against different cell substrates (A-E). The detection of meq antibody was performed with a goat anti-mouse conjugated to Alexa Fluor 488 (Green) and the nuclei stained with Hoechst 33342 (Blue). Labelled cells were observed by fluorescence microscopy. (A) Sf9 cells infected with a MEQ baculovirus. (B) ESCDL-1 cells transfected with pBK-MEQ-CMV. (C) MSB-1 and (D) DT40; for both cell lines, the actin filaments were stained with phalloidin conjugated to Alexa Fluor 594 (red). Meq is detected in the nucleus of all MSB-1 cells indicating that Lamba7 recognizes the Meq protein expressed during latency. As expected, Meq was not detected in DT40 cells. (E) ESCDL-1 cells infected with a recombinant rRB-1B pp38 mcherry. Meq was detected in the nuclei of cells in lytic cycle expressing pp38 mcherry. Bars are shown on each image. [file 13567_2018_526_MOESM1_ESM.tif]

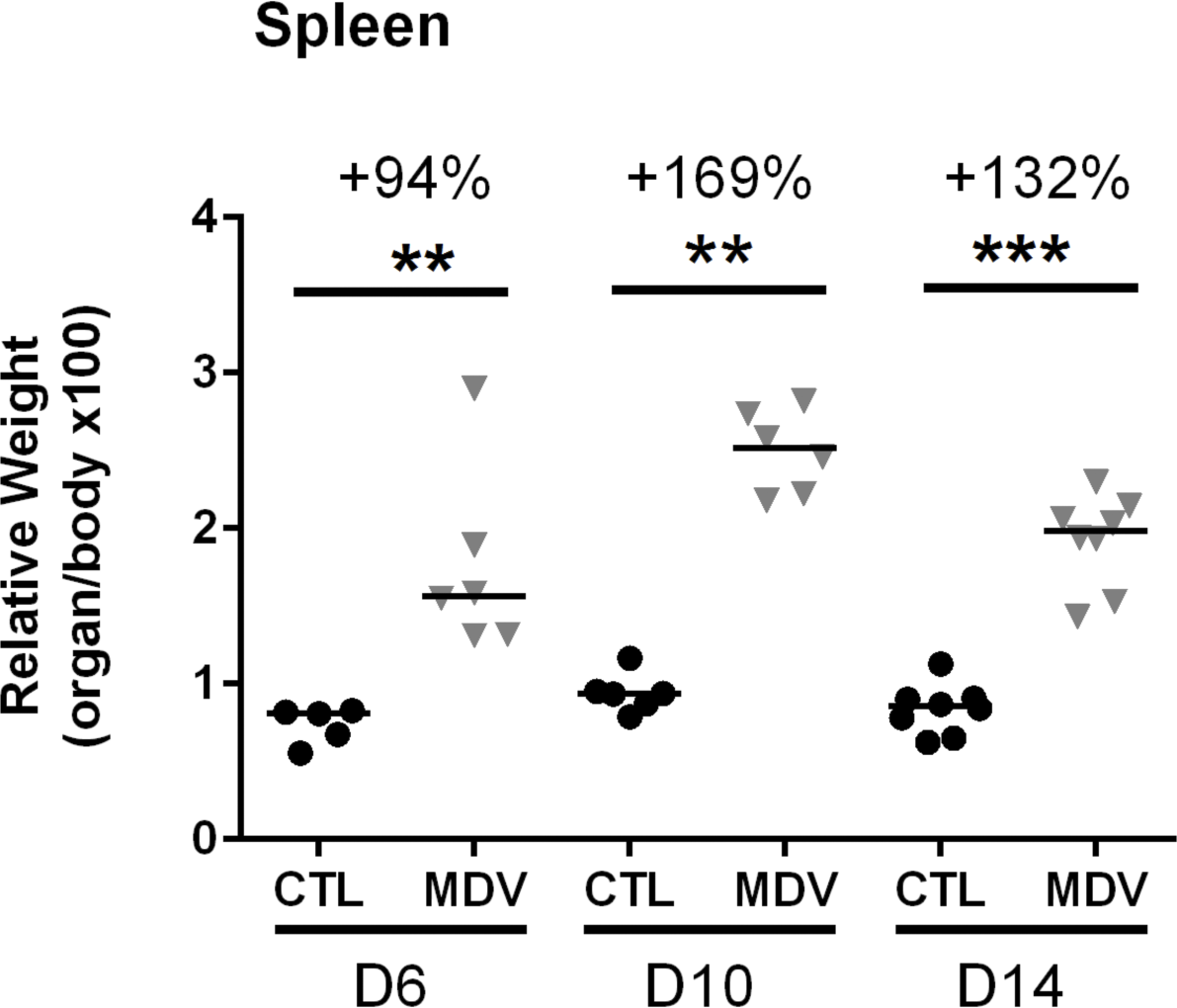

Supplement: Supplementary file 2 — Additional file 2. Splenomegaly induced by the vvMDV RB-1B strain in SPF White Leghorn B19/B19 chicks. Spleen weight/body weight ratios in CTL and MDV-infected chicks at 6, 10 and 14 dpi. The median is represented as a black line. The spleen relative weight was significantly increased from 6 dpi to 14 dpi, in the MDV-infected group compared to the CTL group. [file 13567_2018_526_MOESM2_ESM.tif]
